# Comparison of Fan-Traps and Gravitraps for *Aedes* Mosquito Surveillance in Taiwan

**DOI:** 10.3389/fpubh.2022.778736

**Published:** 2022-03-17

**Authors:** Chao-Ying Pan, Lie Cheng, Wei-Liang Liu, Matthew P. Su, Hui-Pin Ho, Che-Hun Liao, Jui-Hun Chang, Yu-Chieh Yang, Cheng-Chun Hsu, Joh-Jong Huang, Chun-Hong Chen

**Affiliations:** ^1^Department of Health, Kaohsiung City Government, Kaohsiung City, Taiwan; ^2^National Mosquito-Borne Diseases Control Research Center, National Health Research Institutes, Miaoli County, Taiwan; ^3^Institute of Advanced Research, Nagoya University, Nagoya, Japan; ^4^Department of Biological Science, Nagoya University, Nagoya, Japan; ^5^Environmental Protection Bureau, Kaohsiung City Government, Kaohsiung City, Taiwan; ^6^National Institute of Infectious Diseases and Vaccinology, National Health Research Institutes, Miaoli County, Taiwan

**Keywords:** fan-trap, gravitrap, mosquito surveillance, dengue fever vector, mosquito trap, *Aedes*, *Aedes aegypti*, *Aedes albopictus*

## Abstract

A key component of integrated vector management strategies is the efficient implementation of mosquito traps for surveillance and control. Numerous trap types have been created with distinct designs and capture mechanisms, but identification of the most effective trap type is critical for effective implementation. For dengue vector surveillance, previous studies have demonstrated that active traps utilizing CO_2_ attractant are more effective than passive traps for capturing *Aedes* mosquitoes. However, maintaining CO_2_ supply in traps is so labor intensive as to be likely unfeasible in crowded residential areas, and it is unclear how much more effective active traps lacking attractants are than purely passive traps. In this study, we analyzed *Aedes* capture data collected in 2019 from six urban areas in Kaohsiung City to compare *Aedes* mosquito catch rates between (passive) gravitraps and (active) fan-traps. The average gravitrap index (GI) and fan-trap index (FI) values were 0.68 and 3.39 respectively at peak catch times from June to August 2019, with consistently higher FI values calculated in all areas studied. We compared trap indices to reported cases of dengue fever and correlated them with weekly fluctuations in temperature and rainfall. We found that FI trends aligned more closely with case numbers and rainfall than GI values, supporting the use of fan-traps for *Aedes* mosquito surveillance and control as part of broader vector management strategies. Furthermore, combining fan-trap catch data with rapid testing for dengue infections may improve the early identification and prevention of future disease outbreaks.

## Introduction

Female *Aedes aegypti* and *Aedes albopictus* mosquitoes are major vectors of Zika virus, dengue virus, yellow fever, and chikungunya disease (1), all of which lack ideal treatment options. Monitoring of wild mosquito populations and human case numbers is vital for early predictions of disease spread which can facilitate the use of targeted interventions to prevent major outbreaks. A central component of integrated vector management strategies for predicting these trends is mosquito population surveillance *via* trapping (2). Several distinct mosquito traps have thus been developed which implement distinct trapping methodologies (3). These traps can employ different mosquito attractants, such as light, carbon dioxide (CO_2_), and water, to catch female mosquitoes and prevent the occurrence of biting or egg laying in addition to estimating changes in overall population size (4).

Mosquito traps can be stratified into passive or active types, each of which has differing features and degrees of capture efficiencies. Passive traps include ovitraps (4, 5), gravitraps (6), MosquiTRAP™ (7), Mosq-ovitrap (8), Autocidal Gravid Ovitraps (AGO) (9, 10), Gravid Aedes Traps (GAT) (11), and In2Care^®^ Mosquito Traps (12). They do not require a power source for function and rely on lures, such as organic infusions made from dried leaves and grasses, rabbit chow, and green algae, to ensnare mosquitoes (13, 14). Classical ovitrap designs include a lure to attract female mosquitoes to lay eggs inside the trap and mesh to prevent the release of newborns. Gravitraps and other similar traps, including the MosquiTRAP™, AGO, and GAT, build upon this design by including adhesive material in the inner wall of the trap to catch incoming mosquitoes. Passive traps can also be employed to further combat infectious disease. For example, the In2Care mosquito trap includes two distinct toxins: a growth regulator released into the water to kill larvae, and a fungus that rapidly kills infected adult mosquitoes (12). However, while these basic ovitraps and gravitraps are relatively inexpensive and easy to construct, they have low capture efficiency (15).

Active traps require a power source to generate an airflow to capture mosquitoes that fly within their vicinity, which makes them costly and difficult to maintain as compared to passive traps. The BG-Sentinel (Biogents, Regensburg, Germany), Heavy Duty Encephalitis Vector Survey (EVS; BioQuip Products, CA), and Mosquito Magnet Patriot Mosquito (MM; Woodstream Corporation, PA) models improve on basic fan-traps (16) with additional stimuli, such as CO_2_ (*via* fermentation, dry ice, or burning gas) or chemical/air currents, which mimic body-like features to attract female mosquitoes. After capture in these active traps, mosquitoes can be collected *via* airflow or killed by electric shock.

Light traps (17, 18) utilize phototactic stimulation to attract female mosquitoes that exhibit phototaxis, or motility in response to light. Mosquito species, such as *Ae. dupree* and *Culiseta melanura*, are highly attracted to short, or blue, wavelengths of light (19, 20), and this attraction can be influenced with increased light intensity (18). Diurnal mosquitoes, such as *Ae. aegypti* and *Ae. albopictus*, were previously assumed to not be attracted by light (21). However, although *Ae. aegypti* are particularly sensitive to yellow-green light, females from this species are more attracted to red, blue, or purple light when selecting oviposition sites (22).

Given the sizeable differences between trap types, it is unsurprising that capture rates can also vary significantly. For example, a long-term gravitrap *Ae*. *aegypti* index (GAI), or average number of female *Ae. aegypti* caught weekly by each deployed gravitrap, of 0.05–0.3 (23) was calculated from recent studies conducted in Singapore (6). In comparison to active traps, such the BG-Sentinel and CDC light traps, the Mosq-ovitrap showed the lowest capture ability for female *Ae. albopictus* (8.78, 3.15, and 0.78, respectively) (8), demonstrating the sizeable differences in capture efficacy between trap types.

Nevertheless, most studies comparing *Aedes* mosquito capture rates in different traps used CO_2_ (500 mL/min) as an attractant (24–26). However, the use of CO_2_ comes with practical difficulties and costs associated with maintaining consistent gas supplies. It will be therefore be considerably easier and most cost efficient to maintain traps that do not require the use of CO_2_.

The ability to choose the correct trap type and placement is key to monitoring and appropriately responding to disease outbreaks (27). As a real-world example of their use, tens of thousands of dengue fever cases were recorded in the 2014 and 2015 outbreaks in Kaohsiung, Taiwan (28, 29). These outbreaks were influenced by multiple environmental and societal factors (30) and were the impetus for initiation of a new surveillance project to mitigate further outbreaks. From 2017 onwards, the surveillance project included gravitraps for *Aedes* mosquitos. Fan-traps were also installed in distinct locations to identify more efficient methods for mosquito surveillance and capture. Prior to June 2019, Kaohsiung City reported 25 imported and 8 indigenous cases of dengue fever, with seven of those indigenous cases located in the six urban villages around Jinshi Lake. In the entirety of 2019, Kaohsiung City reported 58% of cases in Taiwan were indigenous. Amongst these cases, 46.6% occurred in these six urban villages, which suggests hot spots of dengue fever. Thus, the city prioritized mosquito fogging and puddle removal to reduce the spread of disease. However, it is not known how well the data gathered from the surveillance project reflected the state of this outbreak and therefore what conclusions can be drawn from this project.

In this report, we analyzed and compared catch rates of fan-traps and gravitraps for dengue-associated mosquitoes, *Ae. aegypti* and *Ae. albopictus*, across multiple months in 2019. We found that fan-traps captured greater numbers of *Aedes* mosquitoes (*Ae. aegypti* and *Ae. albopictus*) than gravitraps. We also found that the fan-trap index correlated more closely with reported dengue cases and rainfall levels than the gravitrap index. Thus, mosquito capture data from fan-traps may indicate mosquito population sizes more accurately than gravitraps, and have greater utility for the detection of future disease outbreaks.

## Methods

### Description of Study Area

**Figure 1:**
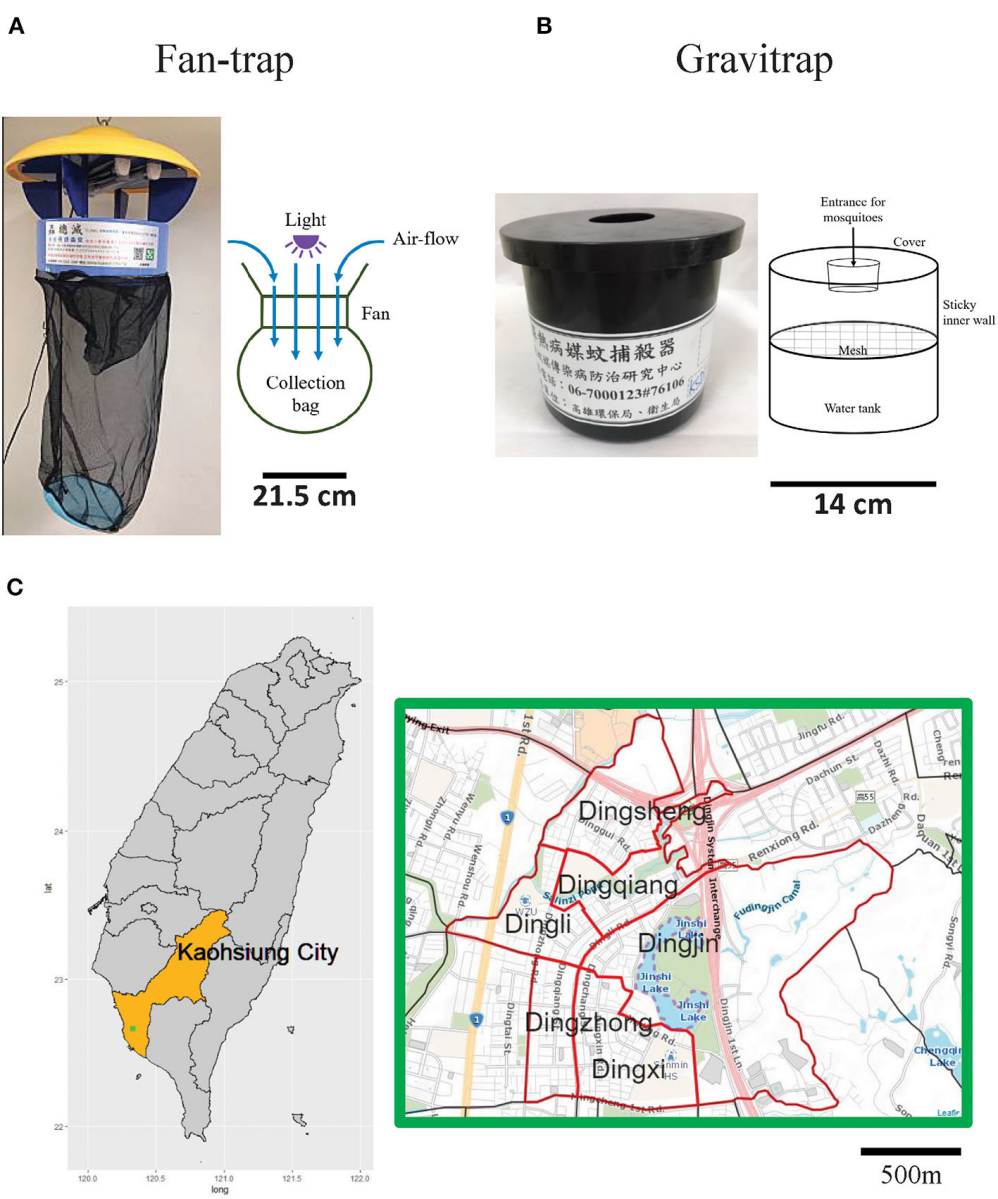
Mosquito traps and surveillance area in this study. Schematic diagrams of a typical **(A)** fan-trap and **(B)** gravitrap. **(C)** Kaohsiung City (left panel) and the six urban villages included in this study (right panel). The geographic location of the six urban villages in Kaohsiung City are denoted by the green dot. The detailed map (right panel) of the six villages located around Jingshi Lake was obtained from https:// https://maps.nlsc.gov.tw/. The placement of mosquito traps is indicated by the red lines, the Jinshi Lake is showed with purple dash line. Maps were created using the R package “Leaflet”.

Field trials were conducted from June to August 2019 in six urban villages, Dingjin, Dingqiang, Dingli, Dingsheng, Dingzhong, and Dingxi, surrounding Jinshi Lake in Sanmin District, Kaohsiung City. This area contains residential, commercial, and park zones. Dingqiang, Dingli, and Dingxi villages have either schoolyards or a campus. Apart from Dingzhong, all the villages have nature water bodies, including lakes, wetlands, and ponds. The study area is 2.88 km^2^ with a population density of over 10,000 people per square kilometer. Kaohsiung City is one of the largest cities in Southern Taiwan (Figure 1C, left panel), with a population of 2.7 million (2019). The average temperature is 26°C, with ~1,800 mm of rainfall per year, providing an ideal environment for *Aedes* mosquito reproduction.

### Mosquito Traps

Fan-traps (e.g., model FL-BW1; Flasco Co., Ltd., Taiwan) and gravitraps (Figures 1A,B) were used in this study. The fan-trap body consists of a multi-wavelength-white-blue double light tube (6 watt), a direct current (DC) motor, and a fan blade, with powered supplied from local buildings. Occasionally, the fan-traps were powered with 6 V lead-acid batteries. The body of the trap was covered with a detachable, beveled-topped plastic lid to prevent electric shock to human users or damage from moving parts. The fan-trap captured insects within the vicinity *via* the fan blade and retained them in a collection cup made of fine mesh. Conversely, the gravitrap lured female mosquitoes for oviposition by providing water at the bottom of the trap. The upper body was composed of a simple black cylindrical trap with adhesive material on the inner surface to capture ovipositing female *Aedes* mosquitoes.

### Trap Positioning

Fan-traps and gravitraps were placed in the study area with a minimum distance of 30 m between traps. Adult mosquito populations were continuously monitored with these traps from June to August 2019. Fan-traps were installed at 1–1.5 m above the ground, while gravitraps were placed on the ground. Data were collected over a 7-day trapping period. Captured mosquitoes from each trap were collected, frozen, and transported to specialists for species and sex identification *via* microscopy of mosquito morphology. The coordinates of all traps were recorded using portable global positioning system (GPS) devices.

### Trap Index Calculation

Mosquito trap indexes were calculated as follows:


$$\begin{array}{l} \;Fan-trap\;index\;\left(FI\right)=\\ \frac{Female\;mosquitoes\left(Ae.\;aegypti+Ae.\;albopictus\right)caught\;by\;traps}{Functional\;Fan-trap\;number},\\ \;Gravitrap\;index\;\left(GI\right)=\\ \frac{Female\;mosquitoes\left(Ae.\;aegypti+Ae.\;albopictus\right)caught\;by\;traps}{Functional\;Gravitrap\;number}. \end{array}$$

The fan-trap index (FI) and gravitrap index (GI) were calculated as ratios of total mosquitoes captured over 3 months to the number of functional traps. A weekly trap index was calculated from mosquitoes captured over a week.

### Statistical Analysis

Statistical analyses were performed using R-4.1.0 (31). The Mann-Whitney-Wilcoxon Test was used for statistical testing with a value of *p* < 0.05 considered as statistically significant for comparisons.

## Results

### Fan-Traps and Gravitraps for Mosquito Surveillance

After 2015, the Kaohsiung City government broadly deployed gravitraps based on Singapore's gravitrap design (6) to dengue fever hot spots for mosquito surveillance. For better attraction of *Aedes* mosquitoes (unpublished data), the design was improved by adding an additional cap with a 5 cm diameter hole to the top of gravitrap, since *Aedes* mosquitoes prefer dark environments for oviposition. We deployed these traps on the ground to attract predominately female mosquitoes in search of locations for oviposition. Conversely, fan-traps (Figure 1A) have been widely used in pig farms in Taiwan to trap Japanese encephalitis mosquitoes (i.e., *Culex tritaeniorhynchus*) (32). These traps were found to catch dengue fever associated mosquitoes and were thus utilized as part of the Kaohsiung mosquito surveillance program. Fan-traps were hung at 1–1.5 m above the ground to match the flight altitude of *Ae. aegypti* and *Ae. albopictus*.

In the first 5 months of 2019, there had been seven reported indigenous dengue fever cases in the study area of Kaohsiung City, which made mosquito surveillance in the six urban villages around Jinshi Lake even more important. To investigate the difference in capture ability between these two often used mosquito traps in Kaohsiung City, we used the actual capture data of the Kaohsiung mosquito surveillance program in this study.

Fan-traps and gravitraps (Figures 1A,B), without CO_2_ bait or lures, were placed in the study area (Figure 1C) for mosquito surveillance with a minimal distance of 30 m between traps to compare catch efficiencies of *Ae. aegypti* and *Ae. albopictus*. During peak time points from June to August 2019, we found that fan-traps cumulatively captured greater numbers of *Ae. aegypti* and *Ae. albopictus* mosquitoes (Table 1). We also observed that fan-traps were able to capture six times more *Ae. aegypti* than *Ae. albopictus*, though fewer *Ae. Aegypti* than *Ae. albopictus* were captured by gravitraps. In both trap types, female mosquitoes were captured more efficiently than males. For example, 81 total female *Aedes* mosquitoes were caught by the gravitraps over the study period, which yielded a GI of 0.68. The fan-traps captured 325 *Aedes* female mosquitoes, giving a FI of 3.39. A total of 99 male mosquitoes were caught by both types of traps. Overall, these data demonstrate that fan-traps were more efficient at capturing *Aedes* mosquitoes compared to gravitraps, regardless of sex.

**Table 1:** Mosquitoes captured by fan-traps and gravitraps (June–August 2019).

**Trap type**	**No. traps deployed**	***Ae. albopictus***		***Ae. aegypti***		**Total**	**Total females**	**Trap index (female)**
		**M**	**F**	**M**	**F**			
Fan-trap	96	16	52	82	273	423	325	3.385
Gravitrap	120	1	42	0	39	82	81	0.675

*M, male; F, female*.

### Weekly Trends Correlated With Captures by Fan-Traps and Gravitraps

**Figure 2:**
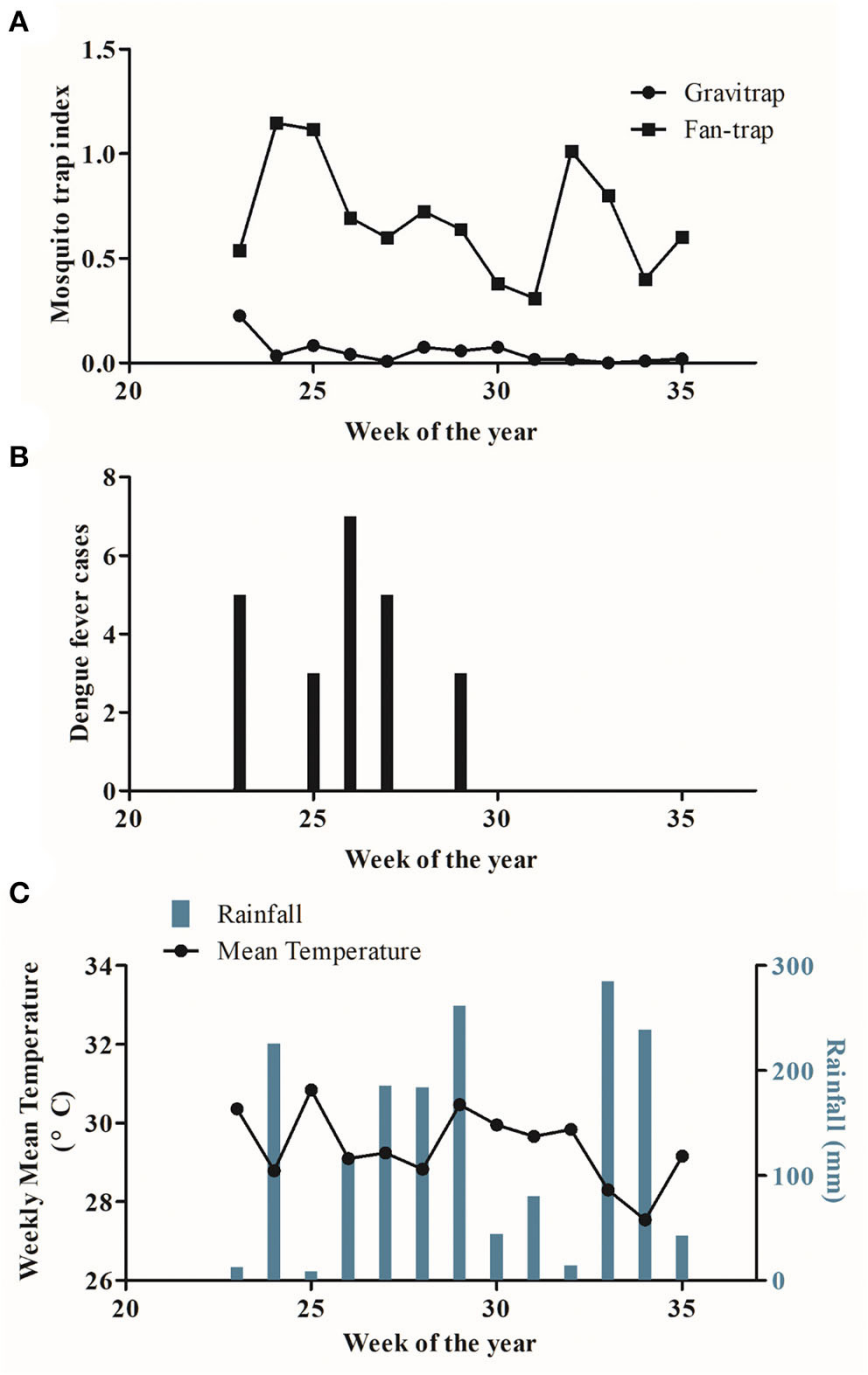
Weekly study data from June to August 2019 in Kaohsiung City. **(A)** Trends of weekly fan-trap mosquito index (FI) and gravitrap mosquito index (GI). **(B)** Dengue fever cases in the study area. **(C)** Average weekly temperatures and rainfall.

**Figure 3:**
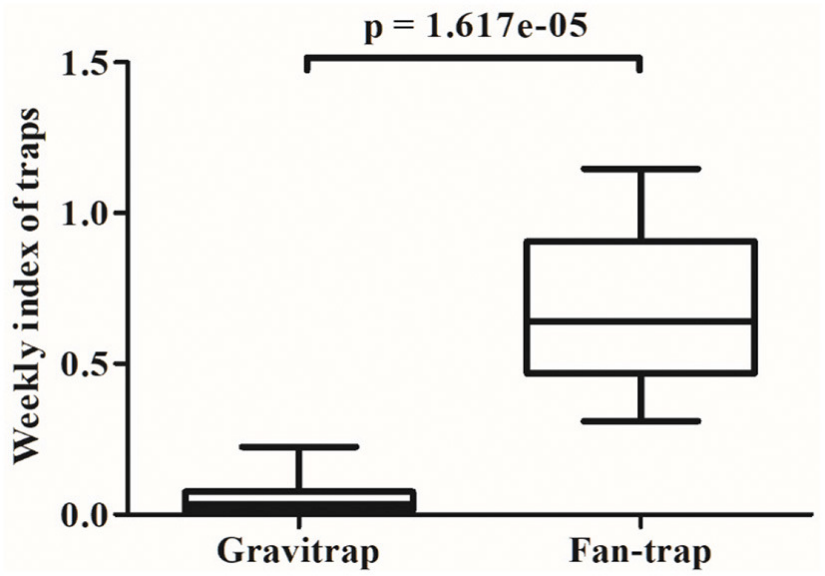
Comparison of weekly fan-trap index (FI) and gravitrap index (GI) values. Statistical analysis of weekly FI and GI values were performed using the Mann-Whitney-Wilcoxon Test (W = 169).

Thus, far, estimates of GI and FI were obtained by averaging the data across the entire time course of the study. We next sought to study weekly variations in FI and GI values. The six villages from this study showed GI values from 0 to 0.23, with an average of 0.05, while the FI ranged from 0.31 to 1.14, with an average of 0.69 (Figure 2A). The GI values peaked around week 23, whereas FI values peaked at weeks 24, 25, and 32. Statistical analysis revealed significant differences between weekly FI and GI (*p* = 1.617 × 10^−5^, W = 169, by the Mann-Whitney-Wilcoxon Test; Figure 3). Furthermore, we also compared the indices of single Fan-traps and gravitraps placed in adjacent locations to check for differences resulting from trap placements. In each of the 8 locations tested we identified greater catch efficiencies for fan-traps than gravitraps; the median weekly FI ranged from 0.33 to 3.88, while the median weekly GI was 0 (Supplementary Figure 1). This suggests distinct *Aedes* mosquito capture capabilities between the two types of traps.

Next, we sought to correlate these peaks in FI and GI with other trends, such as infections and local weather patterns. We correlated capture rates with dengue fever infections by comparing the indices of both traps with locally reported cases from data provided by the Taiwan Centers for Disease Control (Figure 2B). Local dengue fever cases predominantly occurred during weeks 23–29 and peaked at week 26 (seven cases), showing an interesting correlation with peaks in FI at weeks 24 and 25. We found that FI trends were more closely aligned with the pattern of reported dengue fever cases, which showed that fan-traps may be better than gravitraps for the determination of mosquito population sizes. Next, we compared trap indices with local weather data obtained from the Central Weather Bureau, Taiwan. From June to August 2019, the temperature ranged from 27 to 31°C, with over 200 mm of rainfall per week during weeks 24, 29, 33, and 34 (Figure 2C). These temperatures and rainfall provide a highly suitable environment for mosquito reproduction. We found that FI values increased around ±1 on rainy weeks while GI values remained relatively constant. From week 31 onwards, GI values remained very low (0–0.02), which indicated almost no captures by gravitraps during this time.

### Spatial Analysis of Fan-Traps and Gravitraps Show Differences in Capture Efficiency

**Figure 4:**
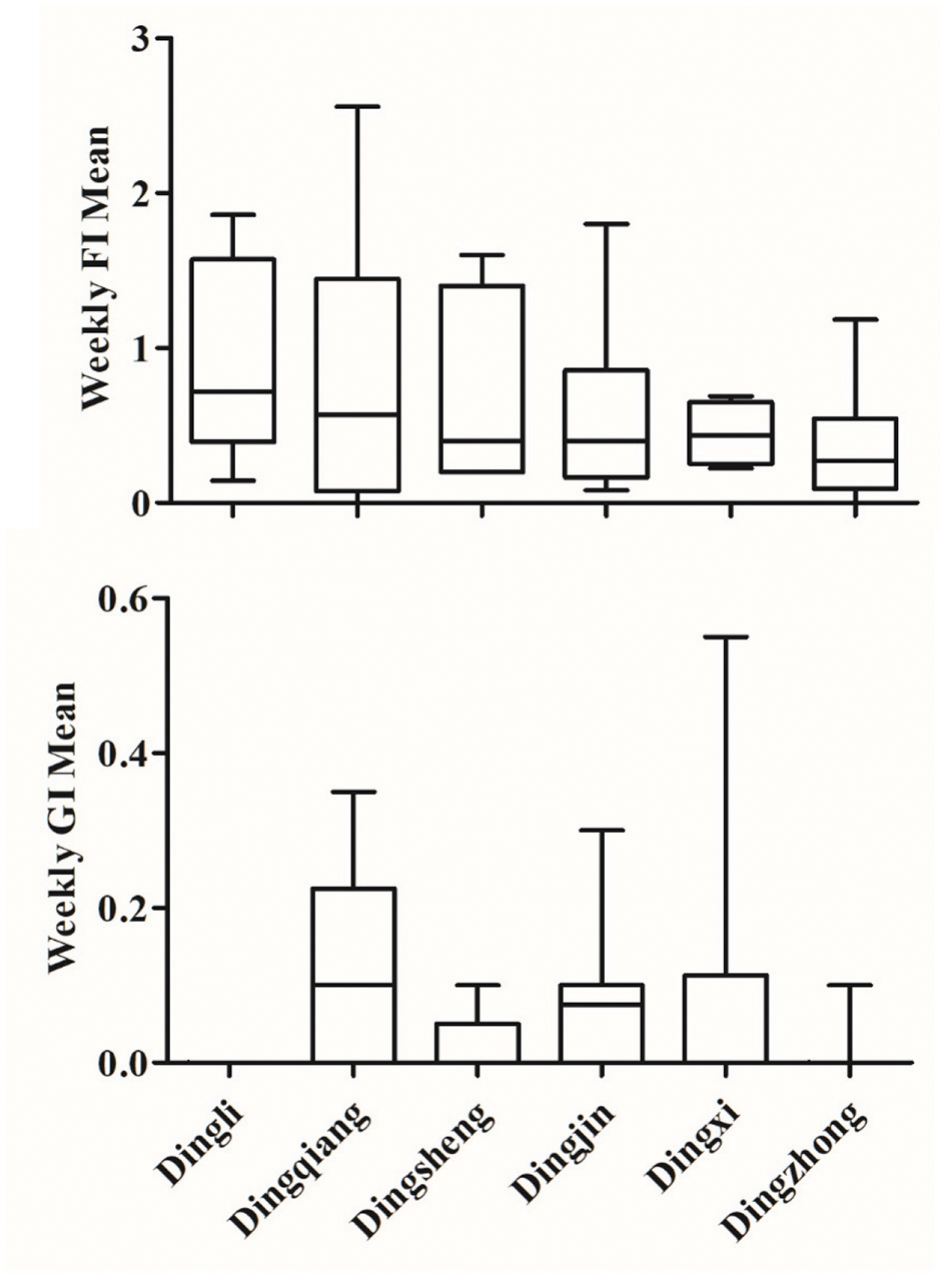
Mean FI and GI values for the six urban villages. calculated FI and GI values for each week are shown in the upper and lower panels, respectively. Data were arranged according to descending mean FI values over the six locations.

We next investigated differences in mean weekly mosquito trap index values across the six urban villages in Kaohsiung City (Figure 4). Dingli had the highest average FI (0.98), while Dingzhong had the lowest (0.37). In contrast, Dingqiang had the highest average GI (0.12) and Dingli had the lowest, with consistent GI values of 0. The inconsistencies between the FI and GI values over the same period strongly demonstrates the differences in capture effectiveness between trap types. The difference in FI could also be due to the difference in overall area of water present (pale blue color in the map of Figure 1C) in each urban village as well as the effects of mosquito control.

## Discussion

Due to the importance of monitoring mosquito populations to prevent and control dengue outbreaks, we here studied the effectiveness of the two mosquito traps currently employed by Kaohsiung City, Taiwan, gravitraps and fan-traps. Though it was known that the two types of traps caught differing numbers of mosquitos and different ratios of female to male, it was not yet known which traps most accurately reflected the status of the local mosquito population. We here show that the fan-traps captured more mosquitos of either sex, and better responded to the changing mosquito population and reported cases of dengue fever.

The selection of the most suitable trap type for any given location requires careful consideration of multiple factors, such as environmental and economic resources. For example, although the cost of gravitraps is much lower than the cost of active traps, gravitraps have repeatedly shown low catch rates (23), including in our data set. Our data analysis supports this conclusion and points to the specificity of these traps for attracting mosquitoes by sex, which is indicated by the capture of 81 females and one male over the 3-month study length. The GI remained under 0.3 female mosquitoes per week from June to August 2019. Although the capture ability of gravitraps can be enhanced with lures, an analysis of their long-term deployment using hay infusion solution in Singapore showed a GAI of <0.3 per week (23). While gravitraps can be enhanced by the addition of chemicals to prevent larval development following oviposition, these methods will be ineffective if the number of female mosquitoes choosing gravitraps remains limited. Another drawback of these traps are their low male capture rates. While males are not disease vectors, their population correlates with the likelihood of female mating and ovipositing. Additionally, male mosquito capture is necessary for the “incompatible insect technique” control programs (33–35), which are based on releasing Wolbachia-infected male mosquitoes to reduce the number of offspring and therefore overall population. Taken together, these data suggests that the capture of *Aedes* mosquitoes with gravitraps may be difficult to optimize and insufficient for proper surveillance.

In this study, we found large differences in absolute FI and GI values as well as trends. When analyzing trap indices and weather records, we noted that the temperatures of the entire study period were in the suitable range for short gonotrophic cycles and high biting rates in mosquito populations (36, 37). Furthermore, weeks 24, 29, 33, and 34 had total rainfall above 200 mm (Figure 2C), which increased FI but caused minimal changes in GI. When comparing trap indices with dengue fever case numbers, the FI values showed closer correlations with these values. After the appearance of dengue fever cases (e.g., weeks 23–29 and 32), the Kaohsiung City government initiated pest control measures, which included insecticide fogging and the removal of standing water in outdoor containers to reduce mosquito numbers. These measures may have resulted in the decreased FI in subsequent weeks.

The fan-traps used in this study were similar to previously used light traps. *Ae. aegypti* and *Ae. albopictus* are diurnal mosquitoes that are infrequently caught by light traps (21) and were largely excluded from light trap research. However, while research suggests that light does not attract *Ae. aegypti* (38), *Ae. albopictus* have been reportedly caught by CDC light traps in greater numbers than by the Mosq-ovitrap (8), which suggest additional factors driving their capture.

We found that more *Aedes* mosquitoes, regardless of sex, were captured by fan-traps than gravitraps, which may be due to their attraction to the sound or air-flow produced by the fan. A newly developed *Aedes* mosquito trap, the male *Aedes* sound trap, uses a 450 or 500 Hz tone as an acoustic lure to capture male mosquitoes (though females are not significantly attracted to these acoustic lures) (35). Alternatively, fan-traps may also create shadows that *Aedes* mosquitoes prefer to stay within. Another major factor to consider is the color of light as an attractant. For example, *Ae. aegypti* showed high oviposition rates when red, blue, or purple light sources were used (22). Therefore, their eyes may be more sensitive to yellow-green and ultraviolet (323–345 nm in wavelength) light, while being less or unaffected by red, blue, or purple light (39), which are factors that may influence their attraction to light traps. The capture of *Ae. aegypti* with autocidal gravid ovitraps (AGO), which uses four tank colors, supports this hypothesis (10). Regardless, it is also possible that mosquitoes are captured solely by the fan without additional attraction.

The six urban villages included in this study represent dengue hot spots in Kaohsiung City, but because the FI values differed between villages, we looked at what may cause this difference and which villages might need more attention in terms of mosquito control. The six villages surround Jinshi Lake, the biggest body of water in our study and likely the major reproductive site for mosquitoes in this area (Figure 1C). We found that Dingli had the highest FI of these six urban villages, which is a proxy of field mosquito populations (Figure 3). This may be attributed to the area of available water in Dingli (pale blue color in the map of Figure 1C), which is more conducive to mosquito reproduction, or the migration of mosquitoes due to pest control measures in the vicinity. The villages Dingqiang and Dingsheng, adjacent to Dingli and containing a wetland and a pond, respectively, had the second and third highest FI. Dingzhong, the only one of these six urban villages without a body of natural water, had the lowest FI. For Dingjin and Dingxi, captured mosquitoes may be lower than expected due to Jinshi Lake being the focal area of mosquito control. Thus, the FI values correlate well with available bodies of water and expected mosquito populations.

By comparing FI and GI values, we found that fan-traps are more efficient than gravitraps at capturing male and female *Aedes* mosquitoes. Our analyses support the use of fan-traps for superior dengue fever mosquito surveillance and control. Although fan-traps require an energy source, they consume low amounts of electricity, which can be readily provided by solar panels and batteries. In urban areas, the electricity can be sourced from nearby houses or streetlamps. As an additional measure for mosquito surveillance programs, captured mosquitoes from the fan-traps can be tested for dengue infection with rapid testing methods (40) to provide more information for dengue disease control, due to correlations previously identified between the number of infected mosquitoes and human dengue fever cases (41, 42). As an additional benefit of fan-traps, previous studies have reported that dengue virus non-structural protein 1, a therapeutic target for dengue virus, can be detected from mosquitoes captured by gravid sticky traps (43). As gravid female mosquitoes begin looking for possible oviposition sites following consumption of a blood meal, individual dengue virus positive females may be more likely to be captured by gravitraps than fan-traps. However, it is difficult to confirm the viral status of females caught in gravitraps due to challenges in retrieving mosquito bodies from sticky trap materials. GI values from these traps are also typically very low, meaning that even if a high proportion of captured females test positive for the virus, the absolute number caught may be less than the number caught by fan-traps.

In comparison, fan-traps are not hindered by retrieval issues, meaning that mosquito bodies can be obtained *via* air-dried conditions (40, 44, 45) and tested for biomarkers of dengue virus. Estimating the efficiency of fan-traps in specifically capturing gravid female mosquitoes should therefore be possible but will require further experiments.

## Conclusion

By comparing the capture ability of gravitraps and fan-traps, which were deployed in Kaohsiung City during the peak of the 2019 dengue fever outbreak, we found that fan-traps caught 4-fold more female *Aedes* mosquitoes than gravitraps, and male mosquitoes were captured at an even higher rate. Weekly analysis clearly showed the difference in capture between the two traps, and the FI seems to be better aligned with the fluctuations of reported dengue fever cases and local rainfall records than the GI. Taken together, our results support the use of fan-traps for dengue fever surveillance.

## Data Availability Statement

The raw data supporting the conclusions of this article will be made available by the authors, without undue reservation.

## Author Contributions

C-YP, LC, J-HC, J-JH, and C-HC: conception and design. LC and MS: formal analysis. C-YP, LC, W-LL, H-PH, C-HL, J-HC, and Y-CY: investigation and data collection. LC, W-LL, MS, and C-CH: methodology. C-HC: funding. LC, W-LL, J-JH, and C-HC: writing—original draft. All authors contributed to the article and approved the submitted version.

## Funding

This study was supported by the National Health Research Institutes (NHRI), Taiwan (grant no. NHRI-MR-110-GP-12) awarded to C-HC. The founders had no role in study design, data collection and analysis, decision to publish, or preparation of the manuscript.

## Conflict of Interest

The authors declare that the research was conducted in the absence of any commercial or financial relationships that could be construed as a potential conflict of interest.

## Publisher's Note

All claims expressed in this article are solely those of the authors and do not necessarily represent those of their affiliated organizations, or those of the publisher, the editors and the reviewers. Any product that may be evaluated in this article, or claim that may be made by its manufacturer, is not guaranteed or endorsed by the publisher.

## Supplementary Material

Supplementary Figure 1 Mean FI and GI values of single trap comparison. Adjacent Fan-trap and gravitrap in eight different locations in the study area were used for comparison. The mean values of weekly FI and GI in each set are shown together. Two sets of single trap comparison in **(A)** Dingjin, **(B)** Dingqiang, **(C)** Dingzhong, and **(D)** Dingxi village were showed.
