# Supplementary figures and images for: Comparison of Fan-Traps and Gravitraps for Aedes Mosquito Surveillance in Taiwan
Source: Front Public Health. 2022 Mar 17;10:778736. doi: 10.3389/fpubh.2022.778736 (PMC8968103; doi:10.3389/fpubh.2022.778736)

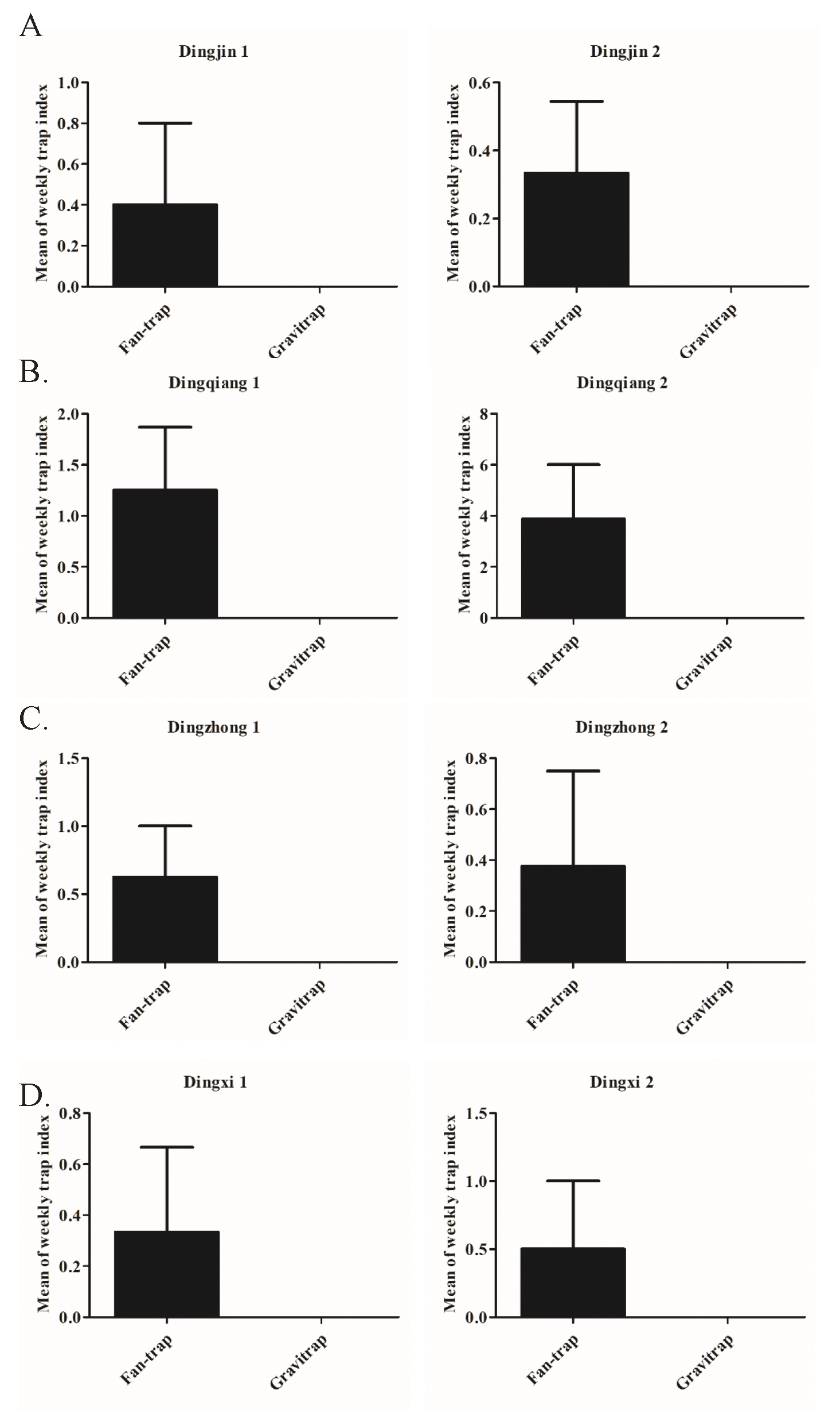

Supplement: Supplementary Figure 1 — Mean FI and GI values of single trap comparison. Adjacent Fan-trap and gravitrap in eight different locations in the study area were used for comparison. The mean values of weekly FI and GI in each set are shown together. Two sets of single trap comparison in (A) Dingjin, (B) Dingqiang, (C) Dingzhong, and (D) Dingxi village were showed. [file Image_1.TIFF]
